# Reviving a Classic Antigen with a Cutting-Edge Approach: Nanobodies for HER2+ Breast Cancer

**DOI:** 10.3390/ph16060794

**Published:** 2023-05-26

**Authors:** Chiara Castrignano, Federica Di Scipio, Francesco Franco, Barbara Mognetti, Giovanni Nicolao Berta

**Affiliations:** 1 Department of Clinical and Biological Sciences, University of Turin, Regione Gonzole 10, 10043 Orbassano, Italy; chiara.castrignano@edu.unito.it (C.C.); federica.discipio@unito.it (F.D.S.); francesco.franco@unito.it (F.F.); 2 Department of Life Sciences and Systems Biology, University of Turin, Via Accademia Albertina 13, 10123 Turin, Italy; barbara.mognetti@unito.it

**Keywords:** nanobody, target therapy, HER2, breast cancer

## Abstract

The serendipitous discovery of nanobodies (NBs) around two decades ago opened the door to new possibilities for innovative strategies, particularly in cancer treatment. These antigen-binding fragments are derived from heavy-chain-only antibodies naturally found in the serum of camelids and sharks. NBs are an appealing agent for the progress of innovative therapeutic strategies because they combine the advantageous assets of smaller molecules and conventional monoclonal antibodies (mAbs). Moreover, the possibility to produce NBs using bacterial systems reduces manufacturing expenses and speeds up the production process, making them a feasible option for the development of new bio-drugs. Several NBs have been developed over the past 10 years and are currently being tested in clinical trials for various human targets. Here, we provide an overview of the notable structural and biochemical characteristics of NBs, particularly in their application against HER2, an extracellular receptor that often gets aberrantly activated during breast cancer tumorigenesis. The focus is on the recent advancements in diagnostic and therapeutic research up to the present date.

## 1. Introduction

Target therapy is a breakthrough strategy slowly paving the way for precision medicine [1]. Essentially, it involves therapeutic methods to target specific pathogenic proteins, cells, or genes within a broader context by exploiting the drug’s ability to bind only the disease-causing molecules while not affecting healthy tissues [2]. Among the medications employed in target treatment, two categories have been principally developed: tyrosine-kinase inhibitors and monoclonal antibodies (mAbs), which are mostly used nowadays [3].

### 1.1. mAbs Limitations in Cancer-Target Therapy

Cancer patients’ clinical care and overall quality of life have both significantly improved thanks to mAbs, as their affinity and specificity for the related antigen allow for more precise treatment and fewer side effects. Jovčevska et al. [4] stated that despite their initial success in the therapeutic field, mAbs have considerable limitations in clinical treatment, including size, an extended half-life in the blood, significant background interference, uneven distribution, and immunogenicity.

mAb-based target therapy’s major disadvantage involves mainly their penetration ability in denser tissues, due to their relatively “large size” (14.2 nm × 8.2 nm × 3.8 nm), corresponding to a molecular mass of ~150 kDa [5]. A recent paper suggests that, in vivo, a gram of solid tumor only effectively receives 0.001–0.01% of the injected mAbs [6]. The observed phenomenon is attributed to the “binding site barrier” effect [7], which proposes that antibodies with the highest affinity for their target antigen bind firmly to the first one they encounter. Consequently, mAbs are unable to reach the tumor core and become trapped at the periphery, resulting in incomplete tumor penetration and suboptimal therapeutic efficacy [8]. mAbs are also sensitive to harsh conditions, denaturing agents, and low pHs, which means that the only way they can be administered is intravenously, subcutaneously, or in an organ-specific way [9].

mAbs currently employed for human diagnostic and therapeutic purposes are usually rodents derived. This raises a critical point since they could eventually provoke an immunogenic response, and, therefore, the production of anti-rodents Abs, thus reducing the therapeutic benefits. The introduction of chimeric and humanized mAbs considerably decreased the immunogenicity percentage from 50–75% to 30% [10].

Large-scale mAbs production requires a high-expanse eukaryotic production system since they are primarily expressed in mammalian cells and characterized by a complex hetero-tetrameric structure with several post-translational modifications. Considering all of the above-discussed factors, it is clear that further progress is needed in the field of therapeutic and prognostic Abs to overcome these technical issues.

### 1.2. NBs Discovery

A serendipitous discovery was made in 1993 when a research team at the Vrije Universiteit of Brussel identified a previously unknown Ab category in some animal species [11]. The study concerned the analysis of sera obtained from healthy dromedaries for other purposes and observed the presence of smaller IgG subclasses that lacked the light chains and the first heavy chain constant domain, and that contributed up to 75% of the total serum IgGs. Two years later, in addition to being identified in the serum of other Camelidae species (camel, llama, dromedary, alpaca, guanaco, and vicuna), these specific Ab fragments were discovered also in shark serum [12].

### 1.3. NBs Mechanism of Production

Camelid NBs are a product of B cells, which are able to generate heavy-chain Abs lacking light chains [4]. The variable domain of the heavy chain, which serves as the binding domain for antigens, is the fundamental component of NBs. The absence of light chains in NBs does not necessarily reduce their binding affinity; indeed, the resulting reduced size can even improve specificity for the target antigen [13]. The production of NBs involves immunization and phage display technology, facilitating the generation of a library of NBs and enabling the characterization of these molecules [14].

Because the cells producing these naturally light chain-deficient Abs are prevalent in blood, a camelid model must be immunized with the antigen of interest before generating NBs in the laboratory. This process lasts about 5 to 10 weeks. It is better to immunize more than one animal to enhance the chances of obtaining NBs against predefined epitopes. Since they are outbred animals, each one will produce a distinct immunological response, yielding a bigger panel of NBs from which to select the best-performing one [15]. As first conducted by Ghahroudi et al. [16] in 1997, drawing a small aliquot of peripheral blood from the immunized camel allows the isolation of effector B lymphocytes from the plasma. The total mRNA is then extracted from the B cells and retro-transcribed into cDNA molecules by performing RT-PCR so that the products themselves can be employed as templates for NB amplification.

Due to their monomeric structure and the absence of post-translational modifications, NBs can be expressed in unicellular systems such as *E. coli*, *S. cerevisiae*, and *P. pastoris* as well as in plants and mammalian cells, leading to a significant reduction in manufacturing costs [6].

### 1.4. Differences between NBs and Abs

**Figure 1:**
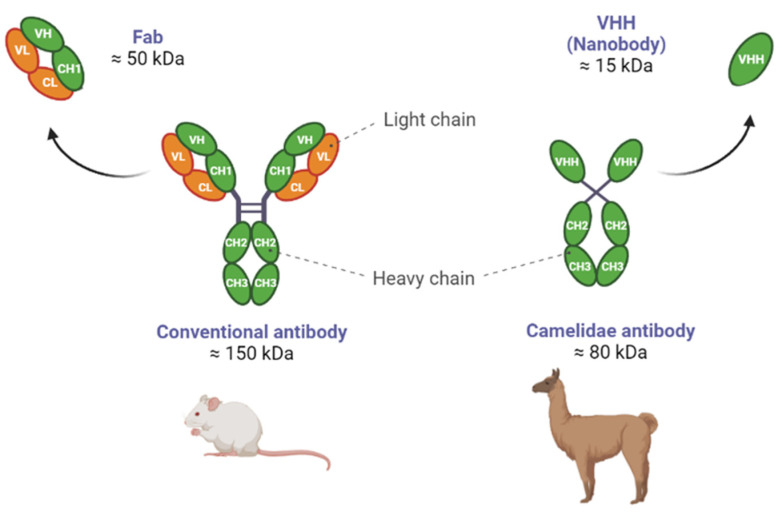
Different antibody structures. Conventional antibodies comprise two identical heavy and two identical light chains associated with disulfide bridges. The antigen-binding fragment (Fab) contains the single-chain variable fragment (scFv), made up of VH and V_L_ domains. The Camelidae antibody comprises two identical heavy chains only. The antigen-binding portion is represented by a single variable region or nanobody (VHH).

These unique single-domain Abs proved themselves to be one of the promising recent innovative breakthroughs that may address mAb deficiencies, as their VHH (Variable domain of Heavy chain of Heterodimeric antibodies) counts only 120 amino acids and a molecular mass of 12–15 kDa. This is the reason why these antigen-binding VHH are also called “nanobodies” (NBs) [17]. NB and other mammalian immunoglobulins VH (Variable domain of Heavy chain) domains, excluding the Camelidae species, have the same structural architecture: three hypervariable antigen-binding loops (FR1–FR4) enclosed by four conserved sequence regions (CDR1–CDR3) [18]. Notably, VHH differs from Abs in that it has two unique structural characteristics. First, compared to the VH domain, VHH has larger CDR1 and CDR3 regions [19]. In particular, the extended CDR3 loop of VHH has more structural flexibility and can penetrate antigen cavities where Abs cannot, thus ensuring an improved binding affinity [20]. The second feature is the abundance of polar and charged amino acids located on the outer part of the molecule, as opposed to the mAbs which possess a larger surface area of non-polar amino acid residues in their structure. The increased frequency of polar and charged amino acids at the solvent-exposed site explains their increased solubility in polar solvents such as water [20]. NBs have an affinity for targets that is equivalent to that of many conventional Abs, with dissociation constants sometimes reaching into the low picomolar range [21]. They have a characteristic prolate shape (4 nm × 2.5 nm × 3 nm) that carries out a full antigen-binding potential and, thus, are the smallest naturally occurring antigen-binding fragments (Figure 1).

The difference between a NB and a human VHH domain is roughly ten amino acids scattered across their sequences: of them, four NB-specific amino acids in the framework-2 region and one in the antigen-binding loops (H3). An Ab variable portion (Fab) is made up of seven amino acids: four in the framework and three in the hypervariable regions. This means that while some VHH signature amino acids can be preserved during the process due to their proximity, others end up being mutagenized in order to obtain a sequence that resembles a human VH the most [22].

Human heavy chain Abs frequently exhibit poor solubility and stability due to a vacant sticky hydrophobic side and a VH domain that lacks a partner. However, camelid VHH domains have the possibility to be humanized. Humanizing NBs for therapeutic applications (i.e., performing an amino acid substitution and replacing hydrophobic residues with hydrophilic ones) is still a common and safer procedure in order to minimize potential immunogenicity and enhance solubility, respectively, by replacing divergent framework residues [23]. A distinctive advantage in the therapeutic use of NBs relies upon the camelid germline immunoglobulin gene and the VH of the human counterpart sequence homology identity, which stands above 80% [24]. Additionally, NBs have low intrinsic immunogenicity because they lack the Fc domain, which might initiate the complement response associated with immunogenicity. Furthermore, NBs can find hidden epitopes that are challenging for Abs to recognize thanks to both their long and flexible CDR3 and their small size, enabling the creation of multivalent NBs for improved therapeutic outcomes (as explained later in this review) [25]. This indicates that NBs will not have a problematic immunogenic profile whatsoever and will thus be safe for human use, as previous studies have proven that repeated NB administration does not cause any humoral or cellular immune response. On the other hand, long-term repeated use of NB-based drugs effects and potential immunogenicity remain to be studied [26].

In contrast to conventional Abs, NBs have a stronger tissue penetration ability and, therefore, can play a significant role in reaching dense tissues. Their half-life in the blood is substantially shorter due to renal clearance as their molecular mass is significantly lower than the renal threshold for glomerular filtration (40–50 kDa). This can be advantageous in circumstances when therapeutic buildup leads to toxicity [26], but it could also represent a problem [4], since depending on the clinical application, it might hardly achieve an optimal target load in vivo.

An advantage of using NBs is their superior resistance properties in harsh conditions, such as pH extremes or chaotropic agents, making them optimal for intravenous, intraperitoneal, and even organ-specific administration.

Overall, both NBs and classical Abs have advantages and disadvantages as therapeutic agents and the choice of which to use depends on the specific application and disease indication (Table 1).

### 1.5. NBs as Enzymatic Modulators

Recent studies have demonstrated that camelid nanobodies (NBs) can act as enzymatic modulators by binding exclusively to the catalytic sites of enzymes, due to the specific recognition capabilities of their paratopes [27,28,29,30]. Enzymatic modulators can be used as therapeutics to treat a range of diseases, including cancer and metabolic disorders [31]. Oyen et al. [32] showed that NBs could be used to modulate the activity of the dihydrofolate reductase enzyme, which is involved in cancer and bacterial infections. A different study, performed in a murine model, also demonstrated the potential effect of the enzymatic modulation of NBs in diagnosing and treating hematological malignancies CD38+, such as chronic lymphocytic leukemia [30]. Since the enzymatic activity of CD38 (Cyclic ADP-Ribose Hydrolase 1) might contribute to a microenvironment favorable for tumor survival in the bone marrow niche, NBs’ tissue penetration capabilities can be exploited to modulate the enzyme activity of CD38 in the bone marrow with a greater likelihood of success.

Overall, NBs’ ability to act as enzymatic modulators opens up new possibilities for the development of novel therapeutics for a range of diseases. However, there are also some challenges associated with the development of such NBs, namely the limited availability of target-specific NBs. In order to modulate enzyme activity, a NB must be able to specifically bind to the enzyme active site. Up to date, a few enzymes have been targeted with NBs and it can be challenging to identify new potential targets [33]. Furthermore, despite the success of some NBs in modulating enzyme activity, the mechanisms by which they work are not always well understood [4].

### 1.6. NBs Extracellular Targets

Thrombotic thrombocytopenic purpura (TTP) was the first pathology to benefit from therapeutic treatment with a NB: in 2019, Caplacizumab (ALX-0681), designed to target the von Willebrand factor, was granted approval by the European Medicines Agency (EMA) and the U.S. Food and Drug Administration (FDA) for the treatment of TTP [34]. The past decades have seen the successful isolation of a large number of NBs that specifically target antigens related to a range of diseases, mostly cancer. NBs have shown intriguing potential in treating solid malignancies: they can be utilized as antagonists inhibiting ligand binding and generating conformational changes that initiate signaling cascades, or as allosteric inhibitors, altering the enzymatic activity of their target proteins [35].

As shown in Table 2 [36], while certain NBs are now being examined in clinical trials for human diagnostic and therapeutic purposes (including approaches involving drug carriers), others have been identified as lead molecules and have moved to advanced preclinical phases.

Extracellular targets, such as soluble factors, ligand receptors, or transmembrane proteins, have been identified as possible targets for NB-based therapeutics thus far. Among the grand range of extracellular receptors usually targeted in cancer therapy, the Human Epidermal Growth Factor Receptor 2 (EGFR2, also known as ERBB2 or HER2) is largely investigated, since its aberrant expression correlates with different malignancies. In approximately 20–25% of breast cancer cases, HER2 is overexpressed due to gene amplification, resulting in elevated levels of the protein on the cell surface [37]. This characterizes a more aggressive behavior, which lowers the likelihood of survival [38]. In physiological conditions, the HER2 receptor is generally activated by homo- or hetero-dimerization and regulates epithelial cell proliferation, differentiation, and survival. Several studies have shown that, when overexpressed, this tumor-associated antigen plays a direct role in tumorigenesis and cancer pathogenesis by activating the mitogen-activated protein kinase (MAPK) and the phosphoinositide 3-kinase (PI3K) pathways [39]. These pathways are both involved in cell differentiation and survival, as well as cell migration. Moreover, HER2+ tumors are prevalently poorly differentiated, as HER2 activation also interferes with cell polarity and adhesion, resulting in an asymmetric overgrowth of more undifferentiated cells [40].

HER2 testing is typically performed as a part of the diagnostic workup for breast cancer, and it is determined by immunohistochemistry techniques. It is utilized as a predictor of mammary tumor behavior and patients with HER2+ breast cancer may be candidates for targeted therapy with HER2 inhibitors. In 1998, after FDA and EMA authorized Trastuzumab, a Copernican revolution occurred: the first humanized mAb was approved for the treatment of metastatic HER2+ breast cancer [41]. It can be used as a single therapy or in combination with other antineoplastic drugs, but patients who received the combined therapy had less tumor recurrence and a better survival rate [42]. Nowadays, besides the surgical and the radiotherapy approach, the pharmacological treatment of breast cancer involves trastuzumab (in case of HER2 positivity), endocrine therapies such as tamoxifen or cytotoxic drugs such as anthracycline, cyclophosphamide, epirubicin and docetaxel, paclitaxel, and carboplatin [43].

Despite advances in breast cancer diagnosis and treatment (and some remarkable new methods on the horizon [44,45]), it remains the primary cause of cancer-related deaths in women worldwide. This is largely due to the fact that tumors have often metastasized by the time of diagnosis [46]. For instance, current imaging methods, such as mammography or magnetic resonance imaging, are not 100% reliable in detecting the early stages of breast cancer because they lack sensitivity [47]. The problem lies in the fact that contrast agents are not tumor-specific; thus, they may distribute in a non-specific way affecting surrounding cells and generating false negatives [48].

In light of what has been explored thus far, and the given NB competitive and unique qualities in research, we decided to focus on their potentiality when used in both imaging strategies and therapy against HER2+ breast cancer.

## 2. NBs for the Diagnosis of HER2

### 2.1. Radioisotope-Based Diagnostic Techniques

Due to its non-invasive nature and ability to offer significant clinical evidence on tumors, molecular imaging (MI) is widely employed in the detection of malignancies [20]. A specific moiety that is to be labeled with a diagnostic radioisotope or an appropriate fluorophore is the principle of this approach [49]. NBs are excellent candidates for MI applications thanks to the above-mentioned properties and their viability and safety have been validated for human use [50,51,52]. In non-targeted organs, with the exception of the kidneys, radiolabeled NBs are typically poorly absorbed, which triggers a high target-to-background ratio soon after administration. As a result, same-day imaging is possible and shorter-lived radioisotopes can be used, which is an improvement over the low target-to-background ratio that is observed quickly after the injection of a full-sized mAb for the same purpose [53]. These properties give rise to NBs’ application in MI methods such as Positron Emission Tomography (PET), Single-Photon Emission Computed Tomography (SPECT), Near Infra-Red (NIR), and Ultrasound-Based Molecular Imaging [54].

**Figure 2:**
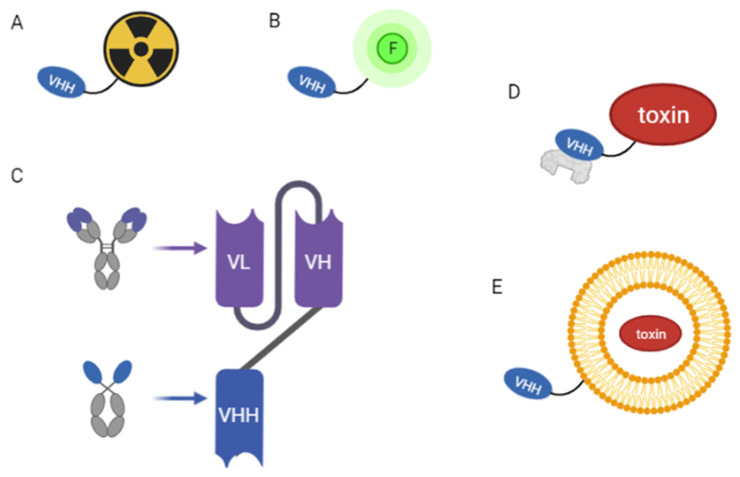
NBs as targeting moieties in diagnostic and therapeutic applications. (**A**) VHH domain conjugated with a radioisotope (^18^F, ^68^Ga, ^99m^Tc) for radiation-based diagnostic techniques. (**B**) VHH domain conjugated with a fluorophore for non-radiation-based diagnostic techniques. (**C**) Bispecific antibody. (**D**) VHH conjugated with an albumin-binding domain and a toxin (NB-drug conjugate). (**E**) VHH domain conjugated with a liposome.

Several anti-HER2 NBs have already been employed: along with ^18^F, ^68^Ga (Figure 2A) was one of the first positron-emitting isotopes to be used for PET NB labeling, since they both had comparably short half-lives (109.8 min and 68 min, respectively), making them especially well-suited for MI [20]. Radioisotope accumulation in tumors is increased by NB labeling, allowing for non-invasive detection techniques sensitive down to a picomolar level [55].

Among the pre-clinical trials, in vivo PET/CT imaging of HER2+ tumor-bearing mice was carried out to demonstrate the suitable radiolabeling qualities for ^18^F [56]. The outcomes were comparable if not even better than those reported with ^68^Ga-conjugated NBs, most likely because of the radioisotope having a slightly longer half-life. Similarly, the use of ^131^I-NBs has demonstrated high specificity and affinity for the targeted antigens, resulting in excellent imaging contrast and sensitivity as assessed by PET. By generating a ^131^I-HER2-NB-conjugated radionuclide, Feng et al. [57] proved that, while both site-specific and random NB-tracer conjugation had comparable tumor targeting abilities, the former resulted in lesser accumulation in normal tissues in breast cancer xenograft mice. Ultimately, in a phase I trial on ^68^Ga-HER2-NB, Keyaerts et al. [50] validated the rapid blood clearance with just 10% of the injected activity still in circulation 1 h post-injection. The conjugate was evaluated on 20 women diagnosed with HER2+ breast cancer, either primary or metastatic. Although there was a significant absorption in the kidneys and liver, all other organs that frequently host primary breast cancer or metastases had very low background levels, resulting in an overall favorable ^68^Ga-HER2-NB biodistribution with minimal to no side effects observed. Because this tumor-targeting strategy revealed promising potential, the evaluation of this tracer advanced to the phase II stage for the assessment of brain metastases detection in breast cancer patients [51].

SPECT, on the other hand, uses γ-emitting radioisotopes; in a pre-clinical study, the ^99m^Tc radioisotope was first used to assess tumor accumulation in mice with breast cancer xenografts [58]. The NB-conjugated tracer accumulated visibly at the tumor site of HER2+ xenograft-bearing mice, yet no tumor localization was detected in HER2-xenografted mouse tumors. Only recently, Zhao et al. [59] developed a ^99m^Tc-labeled anti-HER2 NB to investigate its potential as a brand-new tracer for SPECT/CT evaluation of HER2 expression in breast cancer patients. Ten women with both primary and metastatic cancer participated in an open, non-randomized, first-in-human phase I clinical trial. Again, livers and kidneys were the primary sites of uptake but a very low level of radioactivity persisted in the blood and lungs for the first hour following the injection, which allowed for a better tumor-to-background signal within the following hour. Due to the high activity in the circulatory system, the rapid blood clearance not only enabled the conduction of SPECT imaging at early time points but also helped to lower the likelihood of false-positive results. Furthermore, it was possible to achieve significantly greater signal-to-noise ratios for images taken two hours post-injection due to a rapid tracer reduction in the healthy lung tissue, which increased the contrast of the primary and metastatic cancers in the images. In addition, unlike Ab-derived radiotracers, the ^99m^Tc-HER2-NB tracer is designed not to target the same HER2 epitope as the therapeutic Abs, thus avoiding the effect that the circulating drug might have on tumor uptake. The effectiveness of this NB-radioisotope conjugate will most likely be examined in future studies with larger cohorts.

### 2.2. Non-Radioisotope-Based Diagnostic Techniques

Nonetheless, the risk of radiation exposure for both the patient and the clinician remains a clear drawback of the use of radioisotopes as imaging agents. In theory, isotopes with a short half-life and high positron emission, namely ^18^F, could allow imaging promptly after administration. However, this implies that key parameters such as blood clearance and tissue penetration are compatible with the visibility of the interested target [60]. As a consequence, non-radioisotope-based techniques (Figure 2B) tend to be interesting options, even though they too have drawbacks. Albeit, tissue and body fluids can absorb excitation light and emission wavelengths, fluorescence-based approaches might as well be a viable option. Using appropriately labeled NBs is the only way to perform these specific optic techniques. In their study for the assessment of the anti-HER2 NB drug conjugate, Xenaki et al. [61] performed an experiment on breast cancer-bearing mice xenografts that proved that these alternative strategies can be informative. The signal from HER2+ xenograft mice tumors could be revealed directly by coupling the anti-HER2 NB with a NIR (near-infrared) fluorophore, while still preserving adequate biodistribution. The perspective of a NIR-conjugated anti-HER2 NB could enable the precise non-invasive categorization of HER2+ cancers and more accurate surgical tumor removal in a future therapeutic context [62].

## 3. NBs against HER2 in Therapy

NBs targeting tumor-related receptors (EGFR family, VEGF, c-Met, etc.) are of particular relevance for therapeutic purposes. HER2 is among the most extensively researched oncogenes. Due to its overexpression in many cancers and its limited presence in healthy tissues, HER2 serves as both an oncogene and a good tumor antigen [58]. A single NB directed against HER2 is able to restrain cell mitosis and accelerate cell apoptosis, through the RAS-RAF-MAPK and PI3K-AKT-mTOR pathways. After treatment with an anti-HER2 NB, Western blot analysis of HER2+ breast cell lines (BT474 and SKBR3) revealed that the levels of p-AKT and p-ERK significantly dropped, whereas no fluctuations were observed in cell lines that weakly expressed the receptor (MCF-7) [63].

### 3.1. Identification of NBs against HER2 Extracellular Domain and Tyrosine-Kinase Domain

Approximately a decade ago, the first anti-HER2 NB (SR-87) was successfully isolated using phage display technology. This nanobody exhibited a high affinity for the extracellular domain of the target [64]. The subsequent strategy aimed to explore supplementary prospective domains that could be targeted in order to obstruct any possible divergent pathways that may still be functional or that could trigger a resistance reaction. Since then, a significant amount of research has focused not only on directing HER2 extracellular domain but also on NB binding alternative domains that play a role in the activation of the downstream pathways.

Ten years after the characterization of the extracellular domain-specific NB, Lamtha et al. [65] first managed to isolate a NB specifically directed against the HER2 tyrosine-kinase (TK) domain (VHH17); it had the potential to be further developed into a specific NB that binds and prevents the phosphorylation, therefore, provoking cell death. Starting from a humanized VHH phage library obtained from the venous blood collected from a naïve dromedary, the Ab selection was made using the HER2-TK recombinant protein as the antigen. VHH17 established a binding specifically with the HER2-TK activation loop (CDR1, CD2, and CDR3). The authors successfully managed to show the potential of employing a humanized NB phage display library to create a NB that binds specifically to the ATP-binding pocket and prevents the HER2-TK phosphorylation event. Therefore, this highlights the NB potential for decreasing the cancer cell viability, making it an attractive alternative as a HER2+ therapeutic agent.

However, there have been fewer reports concerning HER2 NBs that exert a suppressive role on HER2+ breast cancer. Whether this interesting approach is effective as an anti-cancer treatment will only be revealed by further studies.

### 3.2. Engineered NBs

#### 3.2.1. Bispecific NBs against HER2

The prospect of designing HER2-targeting therapy for human tumors will be greatly simplified by manufacturing tumor-targeting HER2 NBs. Since 2013, many researchers have attempted to isolate anti-HER2 NBs from immunized Camelidae to first evaluate their efficacy in vitro [66]. The accessibility of these VHH libraries represents a promising prospect for establishing customized protocols that could potentially augment therapeutic efficacy and improve clinical outcomes. Wu et al. [67] originally showed that, similar to what is observed with Trastuzumab, when HER2+ or HER2− cells are treated with their anti-HER2 NB C3 coupled with a human IgG Fc alone (C3-Fc), no tumor cell growth inhibition was seen. On the other hand, when in the presence of NK cells, C3-Fc elicits potent Ab-dependent cell cytotoxicity against HER2+ cells both in vitro and in vivo, even stronger than that attributable to Trastuzumab. According to the epitope mapping investigation, C3-Fc interacts with HER2 extracellular domain through a different epitope than Trastuzumab [68]. This opens up the possibility of employing C3-Fc alone or in combination with another anti-HER2 Ab, delivering an additive or synergistic benefit to the therapy. This kind of construct is known as a “bispecific antibody” (Figure 2C), and it is one of the methods being explored to address Ab resistance [69]. By possessing two distinct antigen binding sites, wherein one recognizes the tumor cells and the other the immune cells (typically T cells or NK cells), they can focus the immune system attention on the tumor cells themselves.

Several anti-HER2 bispecific Ab designs have been examined in the past [70,71,72,73]; however, they come with a number of shortcomings, such as a mixed population after purification, a low yield of production, a predisposition to aggregation, and a brief half-life. Research has recently focused on the potential to create a bispecific Ab that joins a Fab domain against CD3 to a VHH against HER2 or even a Fab domain against HER2 to a VHH against CD16 [72,74]. In this instance, these bispecific Abs can also effectively be secreted and purified in huge amounts from bacterial culture media, reducing the time and manufacturing costs. Once purified, they can exert lethal effects equivalent to, if not greater, than those of the therapeutic mAb alone on tumor cells that specifically overexpress HER2 both in vitro and in vivo, redirecting the T cells or NK cells effects only. With various formats of bispecific Abs targeting HER2+ cancer cells being tested, it will be interesting to observe how they each operate in patient care.

#### 3.2.2. NBs as Drug Carriers

Most often the NB functions as a carrier that is conjugated to a targeting moiety rather than being the drug itself. NB modifications that are intended for conjugation can be carried out without endangering its targeting abilities: this enables the achievement of an effective loading capacity without the need to provide high dosages of the medication [75]. This suggests that, based on the weight of conjugates, NB delivers a considerably higher drug amount with the same drug-antibody ratio value than an Ab-drug conjugate [20]. Drug-loading capacity is defined by the amount of drug loaded per unit weight of vectors, and an NB has a far lower molecular weight than an Ab. Although a high drug-antibody ratio can be used to increase the efficacy of the Ab-drug conjugate itself, it might also decrease the mAb stability and promote drug clearance. Up to date, Trastuzumab emtansine [76] (approved by the FDA in 2013) and Trastuzumab deruxtecan [77] (approved by the FDA in 2022) are the only two clinically authorized humanized anti-HER2 mAbs currently used for treating HER2+ solid tumors. The main drawback of these conjugates, due to the characteristics of the Ab itself, is their heterogeneity in intra-tumoral distribution resulting in sluggish or confined tumor penetration (because of the previously described “binding-site barrier” phenomenon [7]).

#### 3.2.3. Improving Pharmacokinetic Properties

When NBs are intended to be employed as cytotoxic drug carriers, the main drawback is their fast renal clearance, which results in a limited half-life in circulation [26]. Big efforts have been conducted so far to improve their unfavorable pharmacokinetic characteristics. The prospective employment of albumin or albumin domain-binding fusion, as well as PEGylation to increase the size above the glomerular filtration threshold, are all strategies that could extend the in vivo half-life of small therapeutics [78].

Xenaki et al. [61] tested the anti-HER2 NB 11A4 coupled with the albumin-binding domain (Figure 2D) as a possible targeting moiety for NB-drug conjugates’ development. As a result of their investigation, they demonstrated that NBs could serve as a platform for the generation of drug conjugates, providing a more comprehensive effect on tumor targeting. Since lengthening blood half-life is necessary for their in vivo effectiveness, coupling the anti-HER2 NB with an albumin-binding domain can successfully induce protracted and uniform accumulation in the tumor. According to their research, a single dose of an anti-HER2 Auristatin F drug exhibited remarkable efficacy in a pre-clinical model of HER2+ cells. Such long-circulating formulations have been demonstrated to preferentially collect within cancer as a result of the leaky vasculature created by tumors to maintain their growth.

To increase the therapeutic efficacy in HER2+ cancer cells, Farasat et al. [79] showed that a combination of four anti-HER2 NBs targeting various HER2 epitopes could be coupled on doxorubicin-loaded PEGylated liposomes (Figure 2E). Because of the intrinsic specificity of NBs, the nano-complexes in this scenario were able to bind to HER2-overexpressing tumor cells, leading to a higher toxicity rate and fewer side effects. Similarly, polymeric nanoparticles gained popularity as nanocarriers over the past two decades thanks to their capacity to increase the physicochemical stability of their payload along with fairly good control over their release.

Along the lines of differently loaded PEGylated liposomes, Martínez-Jothar et al. [80] employed the same PEGylation approach to create a nanoparticle capable of encapsulating and delivering saporin, a novel anti-neoplastic agent inducing DNA breaks and apoptosis. By equipping the nanoparticle outer layer with NBs able to bind to cell surface receptors that are overexpressed in tumors, efficiency and selectivity were guaranteed. For this study, an 11A4 NB directed against HER2 was used to improve the polymeric NPs’ selective uptake by HER2+ breast cancer cells (SKBR3) in comparison to the HER2-cells (MDA-MB-231). After nanoparticle endocytosis occurred, the photochemical internalization approach was employed to trigger ROS production upon excitation with light of the proper wavelength; in this way, the endosomal membrane gets damaged and allows the release of the contained saporin into the cytosol. The selective NB-mediated endocytosis combined with the photochemical internalization enables the spatiotemporally and regulated release of the nanoparticles and their cargo from the endosome, consequently exposing the cells to saporin-damaging effects. A parallel approach may be employed to load therapeutic compounds into extracellular vesicles that overexpress anti-HER2 NBs on their surface [81]. This emerging drug delivery system is a natural means of eukaryotic intercellular communication, meaning that the ability to bypass cellular membranes can be exploited in non-immunogenic therapies.

This effectively demonstrates the versatility of an NB-based approach, prompting further testing in various types of cancer models as well NB-drug conjugates benefitting from enhanced drug solubility, circulation, decreased immunogenicity, and controlled release simply by undergoing a conjugation with a carrier molecule such as albumin or PEGylated particles.

#### 3.2.4. NBs in Radiopharmaceutical Therapy

Current and proposed cancer treatment approaches in radiopharmaceutical therapy (RPT) include targeted radionuclide therapy, where the energy absorbed from radiation released by radionuclides with short path lengths (α- or β-particles, and Auger electrons) produces a biological effect [82]. By combining with high-specificity carriers (i.e., Abs [83]) or through physiological uptake, therapeutic radionuclides can build up in lesions of interest. RPT has emerged as an appealing cancer therapy option, particularly for individuals with metastatic disease, including breast carcinomas with HER2 overexpression [84]. For RPT to work, a radioactive atom and a tumor-targeting vector must be properly combined and the two must be linked in the right way. With fewer adverse effects than might be experienced with conventional radiotherapy, this ensures the possibility of increasing patient survival. It is, therefore, worth mentioning the prospective employment of NBs in RPT that has been evaluated during the last few years [85]. Feng et al. [86] conjugated the VHH_1028 anti-HER2 NB with a ^131^I-labeled prosthetic agent and its tissue distribution was analyzed in a murine HER2+ breast cancer xenograft model. The comparison was performed with another HER2-targeted radiopharmaceutical being tested in clinical trials ([^131^I]SGMIB-2Rs15d); 2Rs15d was the first anti-HER2 NB used as a targeted radionuclide agent for the treatment of HER2+ breast cancer, with tumor targeting, biodistribution, and safety evaluated in a pre-clinical investigation before moving on to a phase I clinical trial [87].

The results showed that iso-[^131^I]SGMIB-VHH_1028 exhibited 6.3 times more beneficial tumor-to-kidney radiation dosage ratios. Multiple-dose therapy regimens revealed that the compound was well tolerated, significantly slowed tumor development, and extended survival.

After successfully developing the ^99m^Tc-NM-02 MI tracer with NM-02 NB as the lead compound [59], Zhao et al. recently investigated whether labeling said NB with ^131^I may be employed as a targeted radionuclide treatment agent for HER2+ breast cancer in xenografted mice [88]. One of the main reasons ^131^I was employed in this work is because its theranostic properties, namely the ability to emit β-particles and γ rays, enable it to perform both SPECT imaging and radionuclide therapy [89]. In comparison to mice with HER2- tumors, ^131^I-NM-02-treated HER2+ xenografts displayed significantly reduced tumor growth and increased survival times with optimal organ compatibility, hinting at the potential translation of said agent for clinical use [88].

Targeted α-particle therapy has also emerged as an appealing approach in RPT since it is able to exert cytotoxic effects via processes distinct from those used by already approved HER2-targeted pharmaceuticals. The use of α-particles makes it possible to irradiate metastases while causing the least possible damage to the nearby healthy tissues. HER2-targeted Abs were labeled with a range of α-emitters and their therapeutic potential was assessed in both animal models and patients [90]. Nevertheless, issues arose with the administration of such Abs, which was delayed and uneven. To overcome these obstacles, Feng et al. [91] substituted Abs with NBs as alternate scaffolding for targeted anticancer therapy, with a focus on the HER2-targeting moiety. In a prior work, they found that combining the prosthetic agent ^211^At labeling and the anti-HER2 NB properties might lead to an improved tumor targeting [92]. With this most recent study [91], they also verified the therapeutic efficacy of ^211^At-labeled NB conjugates, along with a long-lasting dosage-dependent effect following a single dose administration in a xenograft model for subcutaneous HER2+ breast cancer. Although further research into this targeted α-particles technique for the treatment of patients with HER2+ malignancies is required, the fast and even tumor penetration properties of NBs have once more come to light as desirable traits for yet another cancer target treatment.

Considering the preclinical results produced thus far, the utility of these NB tracers as theranostic agents has been widely recognized.

## 4. Conclusions

The research for the optimal breast cancer treatment is still working on designing new strategies. Gene therapy has been researched as a potential treatment option, though it is still in the early phases of development [93]. Viral vectors are typically used for gene delivery. However, since adverse effects including immunological responses and even mortality have been reported, research has since moved towards non-viral vectors, such as liposomes [94]. The significant question of how to successfully deliver the therapeutic gene to the intended region is one of a number of challenges that prevent gene therapy from progressing faster. These obstacles also include mutagenesis, the inability to treat tumors that are inaccessible, and many others [95]. A way to overcome the problem of gene delivery can be solved by employing nanoparticles, as they protect genetic tools from degradation and promote their accumulation in tumor cells, boosting efficacy in gene expression regulation, while decreasing breast cancer progression [96]. Another strategy using gene therapy entails employing tools for direct DNA modification, such as CRISPR/Cas9, either to silence genes that promote cancer growth or to improve the immune system’s capacity to recognize and eliminate cancer cells [97]. Despite one successful HER2-targeting study [98], CRISPR/Cas9 genome editing is known to cause both genotoxicity and cytotoxicity, as well as an assortment of possible off-target effects. Although some viral vector-gene therapies have already been approved [99,100,101,102,103,104], none have successfully targeted cancer, particularly breast cancer.

NBs’ big breakout relies upon their advantageous features, including higher specificity, lesser toxicity, and improved safety, which are highly regarded in the oncology field. Along with the optimal in vitro reproducibility and relatively low manufacturing costs of the phage display technology, humanized NBs against specific tumor-driving and tumor-associated antigens can be easily developed.

These properties make them suitable carriers for MI and therapy. They do, however, have their own limitations for therapeutic uses, namely rapid renal clearance which prevents a high load on the diseased tissue and causes kidney toxicity. Nevertheless, strategies for engineering them into constructs with higher efficacy and fewer undesirable effects are being developed. Given these considerations, approaches for NB functionalization and delivery establish a solid platform for the development of advanced diagnostic and therapeutic tools.

NBs have driven significant advances in diagnostic research, with multiple clinical trials at stages I and II currently ongoing, yet their application in clinical settings is relatively little explored, with investigations mainly up to pre-clinical trials. This enables further investigation of the limitless potential of NBs and positioning them as pharmaceuticals for use in routine clinical practice. It is not unlikely that improved drug delivery methods might aid in the success of NBs over antibodies in clinical care.

## Figures and Tables

**Table 1:** Advantages and disadvantages of Abs and NBs.

	Advantages	Disadvantages
**Abs**	Already approved as therapeutics; Established safety and efficacy; High loading capacity; Trigger the immune response to eliminate the target antigen; Requires mice for the immunization process.	Limited tissue penetration due to large size; Higher production requirements; Sensitive to harsh conditions; Limited administration routes; Immunogenicity.
**NBs**	High solubility; High penetration capabilities due to small size; Easy to generate; Highly stable; Low immunogenicity.	Limited availability; Short half-life; Limited understanding of the mechanism of action due to the novelty; Requires camelids/sharks for the immunization process.

**Table 2:** Currently undergoing clinical trials for NBs, both diagnostic and therapeutic.

Condition/Disease	Target	NB Drug	Clinical Trial	Phase	Study Start
Hidradenitis Suppurativa ^1^	IL17A, IL17F, IL17A/F	M1095	NCT05322473	II	April 2022
Generalized Myasthenia Gravis ^2^	AChR	ALXN1720	NCT05556096	III	November 2022
Solid tumor PET/CT ^3^	PD-L1	^68^Ga-THP-APN09	NCT05156515	NA	December 2021
Solid Tumor PET/CT ^4^	CLDN18.2	^18^F-FDG	NCT05436093	NA	June 2022
Non-small-cell lung cancer, mesothelioma, colorectal cancer ^5^	MSLN	α-PD1-MSLN-CAR-T cells	NCT05373147 NCT05089266	Early I I	October 2020 November 2021
Malignant lymphoma ^6^	IL21	JS014	NCT05296772	I	February 2022
Malignant Neoplasm of Digestive System ^7^	CLDN18.2	DR30303	NCT05639153	I	May 2022
Cancer SPECT/CT ^8^	HER2	^99m^Tc-MIRC208	NCT04591652	II	April 2019
Metastatic breast cancer PET/CT ^9^	HER2	^68^GaNOTA-anti-HER2 VHH1	NCT03331601 NCT03924466	II II	October 2017 April 2019
Malignant solid tumor PET/CT, Cardiovascular atherosclerosis, Lymphoma, Cardiac Sarcoidosis ^10^		^68^Ga-NOTA-anti-MMR VHH2	NCT04168528 NCT04758650	I–II II	November 2019 January 2021

^1^ Interleukin-17-targeting M1095 (sonelokimab, previously used in the phase I trial for the treatment of psoriasis) administered subcutaneously compared with a placebo in the treatment of adult participants with moderate to severe hidradenitis suppurativa. ^2^ ALXN1720 for the treatment of generalized Myasthenia Gravis in adults with autoantibodies against acetylcholine receptor. ^3^ Non-invasive approaches using ^68^Ga-THP-APN09 PET/CT to detect the PD-L1 expression of tumor lesions in patients with lung cancer, melanoma, and other solid tumors to identify patients benefiting from anti-PD-(L)1 treatment. ^4^ Non-invasive approach ^68^Ga-ACN376 PET/CT to detect the CLDN18.2 expression of tumor lesions in patients with solid tumors and to identify patients benefiting from CLDN18.2 targeting treatment. ^5^ Trial that assesses tolerability of autologous mesothelin (MSLN)-targeted chimeric antigen receptor (MSLN-CAR) T cells secreting PD-1 nanobodies (αPD1-MSLN-CAR T cells) in patients with solid tumors. ^6^ BOIN design to assess the safety and potential efficacy of JS014 at different dose levels as a single agent and in combination with a fixed dose of pembrolizumab in subjects with advanced cancer. ^7^ CLDN18.2-targeting DR30303 is tested against advanced and/or metastatic solid tumors. ^8^ Whole-body SPECT/CT study to investigate the clinical value of ^99m^Tc-MIRC208 in HER2 status measurement of cancer patients. ^9^ To quantify the uptake of 68GaNOTA-Anti-HER2 VHH1 in local or distant metastases from breast carcinoma patients using PET/CT imaging and to assess the repeatability of the image-based HER2 quantification. ^10^ Evaluation of the ^68^NOTA-anti-MMR-VHH2 uptake for in vivo imaging of Macrophage Mannose Receptor (MMR)-expressing Macrophages by means of PET/CT imaging of breast cancer, head and neck cancer, and for PET in patients with oncological lesions in need of non-surgical therapy, patients with cardiovascular atherosclerosis, syndrome with abnormal immune activation and cardiac sarcoidosis.

## Data Availability

Not applicable.
